# Editorial: The Evolution of Rhythm Cognition: Timing in Music and Speech

**DOI:** 10.3389/fnhum.2017.00303

**Published:** 2017-06-13

**Authors:** Andrea Ravignani, Henkjan Honing, Sonja A. Kotz

**Affiliations:** ^1^Veterinary and Research Department, Sealcentre PieterburenPieterburen, Netherlands; ^2^Language and Cognition Department, Max Planck Institute for PsycholinguisticsNijmegen, Netherlands; ^3^Artificial Intelligence Lab, Vrije Universiteit BrusselBrussels, Belgium; ^4^Music Cognition Group, Amsterdam Brain and Cognition, Institute for Logic, Language, and Computation, University of AmsterdamAmsterdam, Netherlands; ^5^Basic and Applied NeuroDynamics Lab, Faculty of Psychology and Neuroscience, Department of Neuropsychology and Psychopharmacology, Maastricht UniversityMaastricht, Netherlands; ^6^Department of Neuropsychology, Max-Planck Institute for Human Cognitive and Brain SciencesLeipzig, Germany

**Keywords:** rhythm, meter, synchrony, interval timing, time perception, beat perception, evolution of speech, evolution of cognition

## Overview of this paper

This editorial serves a number of purposes. First, it aims at summarizing and discussing 33 accepted contributions to the special issue “The evolution of rhythm cognition: Timing in music and speech.” The major focus of the issue is the cognitive neuroscience of rhythm, intended as a neurobehavioral trait undergoing an evolutionary process. Second, this editorial provides the interested reader with a guide to navigate the interdisciplinary contributions to this special issue. For this purpose, we have compiled Table 1, where methods, topics, and study species are summarized and related across contributions. Third, we also briefly highlight research relevant to the evolution of rhythm that has appeared in other journals while this special issue was compiled. Altogether, this editorial constitutes a summary of rhythm research in music and speech spanning two years, from mid-2015 until mid-2017.

**Table 1:** Papers in this issue categorized along methodological and conceptual dimensions.

**Authors**	**Type**	**Animal/Species**	**Method**	**Modality**	**Domain**
Abboub et al.	Original research	Human infant	Behavioral preference	Auditory	Speech, meter
Bedoin et al.	Original research	Human child	Behavioral, operant	Auditory	Music, language
Bekius et al.	Original research	Human adult	Behavioral, operant	Auditory	Music, speech
Benichov et al.	Mini review	Zebra finch	Behavioral and neural	Auditory	Music, speech, song
Celma-Miralles et al.	Original research	Human adult	EEG	Visual, auditory	Music, meter
Cirelli et al.	Original research	Human infant	EEG	Auditory	Music, meter
Cumming et al.	Original research	Human child	Behavioral, operant	Auditory, motor	Music, speech
Dufour et al.	Original research	Chimpanzee	Behavioral recordings	Auditory, motor	Music
Forth et al.	Hypothesis and theory	Human	Quantitative modeling	All	All
Gamba et al.	Original research	Lemur	Behavioral recordings	Auditory	Music, speech, song
Geambaşu et al.	Data report	Human adult	Behavioral, operant	Auditory	Music, speech
Hannon et al.	Original research	Human	Behavioral recordings	Auditory	Music, speech, song
Hartbauer and Römer	Hypothesis and theory	Insects	Behavioral and neural	Auditory, motor	All
Hoeschele and Bowling	Original research	Budgerigar, human	Behavioral, operant	Auditory	Music
Jadoul et al.	Original research	Human adult	Behavioral recordings	Auditory	Speech
Lense and Dykens	Original research	Human	Behavioral, operant	Auditory	Music
Matthews et al.	Original research	Human adult	Behavioral, operant	Auditory, motor	Music, meter
Norton and Scharff	Original research	Zebra finch	Behavioral recordings	Auditory	Music, speech, song
Polak et al.	Original research	Human cross-cultural	Behavioral recordings	Auditory	Music, meter
Rajendran et al.	Original research	Human adult	Behavioral, operant	Auditory	Music, noise
Ravignani et al.	Perspective	Seal, sea lion	Behavioral recordings	Auditory	Music, speech, song
Richter and Ostovar	Hypothesis and theory	Human	Behavioral recordings	Visual, auditory, motor	Music, dance
Roncaglia-Denissen et al.	Original research	Human adult	Behavioral, operant	Auditory	Music, speech
Rouse et al.	Original research	Sea lion	Behavioral, operant	Auditory, motor	Music
Sameiro-Barbosa and Geiser	Mini review	Human	Behavioral and neural recordings	Auditory, motor	Music, meter, dance
Spierings and ten Cate	Opinion	Zebra finch	Behavioral and neural	Auditory	Music, speech, song
Su	Original research	Human	Behavioral, operant	Visual, auditory, motor	Music, meter, dance
Teie	Hypothesis and theory	Human fetus	Behavioral recordings	Auditory	Music, meter
Teki	Mini review	All	All	All	All
Teki and Griffiths	Original research	Human adult	MRI	Auditory	General timing
Teki and Kononowicz	Commentary	Human	Neural recordings	Auditory	Music, meter
ten Cate et al.	Original research	Zebra finch, budgerigar	Behavioral, operant	Auditory	Music
Woolhouse et al.	Original research	Human adult	Behavioral preference	Visual, auditory, motor	Music, dance

## Timing in music and speech

Human speech and music differ in many respects but also share similarities. One of the main similarities lies in their temporal nature. In fact, both music and speech:

- develop over time and have a temporal dimension crucial to *physically* characterize music and speech,
- rely on timing as one of their most conspicuous *perceptual* dimensions,
- can be tokenized as *sequences* of temporal intervals, which are perceived as a “rhythm,”
- are composed by temporal intervals that possibly differ in duration and acoustic marking by different spectral properties, generating *metrical* expectations.

Humans seem to be particularly rhythmic animals. Decades of research have shown that human brains are tuned-in to the fine degrees of rhythmic information in music and speech (Bolton, 1894; Fraisse, 1981, 1982, 1984; Longuet-Higgins and Lee, 1982, 1984; Povel, 1984, 1985; Essens and Povel, 1985; Povel and Essens, 1985; Shmulevich and Povel, 2000). This human propensity to perceive, produce, and process rhythm is increasingly well understood, though its evolutionary origins remain a bit of a mystery. Let's compare this to what we know about the eye. This organ has evolved in animals as a complex photoreceptor to supply the need of sensing light (Fitch, 2015a). In addition, color vision in humans and many other species appears particularly useful to assess the ripeness of food or the quality of a potential mate, hence conferring an evolutionary advantage. Unfortunately, we are still far from providing similar answers for a complex neurobehavioral trait such as rhythm. However, we firmly believe that rhythm needs to be anchored in an evolutionary perspective.

A number of critical questions spurred this special issue. When did the sensitivity for rhythm arise in human evolutionary history? How did rhythm cognition develop in human evolution? How does this evolutionary path relate to rhythm ontogeny? What is the biological function of rhythm in the millisecond to second range? Do environmental rhythms affect the evolution of brain rhythms, and how? Do speech and music share rhythm-specific neural circuits and cognitive modules? Are these circuits shared with other domains and even across species?

## Rhythm: A multidisciplinary field

In general, the long-time scales involved in evolutionary processes prevent direct observation. Sometimes the evolutionary dynamics of simple traits can be replicated in the lab: For instance, the evolution of learning in fruit flies can be directly observed (Mery and Kawecki, 2002). Instead, the evolution of human behavior and neurobiology requires a more indirect scientific method. This is why understanding the cognitive neuroscience of rhythm and its evolution calls for a tight integration of different perspectives (Fitch, 2015b; Honing et al., 2015; Ravignani, 2017a). In particular, complementary approaches include but are not limited to:

1. developmental studies of rhythm that are useful in understanding whether rhythm perception and production involve critical acquisition periods, or instead result mostly from enculturation during the whole lifespan (Hannon and Trehub, 2005),
2. comparative and cross-cultural studies of rhythm that serve to explain whether musical enculturation or exposure to specific languages can affect which specific rhythmic patterns can be produced/perceived and how frequently (Greenberg et al., 1978; Rzeszutek et al., 2012),
3. comparisons of rhythm processing in music and speech, at both behavioral and neural levels (Peretz et al., 2015) that help understanding whether common music-speech networks exist and similar behavioral patterns can be observed when humans engage in music and speech production,
4. evidence and comparison of rhythm processing across modalities and domains that are used to understand whether, for instance, metrical expectation in speech is strictly bound to the speech domain or instead recruits the same capacities for metricality available in music, or even in dance and vision (Iversen et al., 2015),
5. studies of rhythm in interaction and context (Yu and Tomonaga, 2015), explaining how social, affective, and other factors affect the emergence of rhythmic patterns,
6. archaeological findings trying to reconstruct rhythm-related behavior and cognition in our early hominid ancestors (Morley, 2003),
7. mathematical and computational models (e.g., connectionist, symbolic) of the mechanisms underlying perception and production of rhythmic behavior (Desain and Honing, 1989, 1991, 2003),
8. mathematical and computational models of rhythmic capacities as evolved behaviors (Miranda et al., 2003) in line with a long tradition in evolutionary and theoretical biology,
9. evidence of spontaneous rhythmic behavior in other animals (Fuhrmann et al., 2014; Ravignani et al., 2014a) showing how similar rhythmic traits can evolve via similar pressures in phylogenetically distant species,
10. controlled experiments in non-human animals (Cook et al., 2013) probing the potential for producing/perceiving rhythm (even though these are not usually part of these species' natural behavior); these experiments can show the existence of basic, evolutionary conserved cognitive processes that may have been exapted in humans for rhythmic purposes.

The cognitive and neurobiological bases of rhythm are increasingly well understood (Honing et al., 2015; Merchant et al., 2015). While researchers in many fields are interested in rhythm, there is little awareness of how related and potentially converging their research strands are. This special issue builds a bridge across a large number of scientific disciplines; the focus lies in the cognitive neurosciences of rhythm, conceptualizing rhythm as a neurobehavioral trait undergoing an evolutionary process.

## Development evidence

A good proportion of the papers in this issue deals with developmental aspects of rhythm (Abboub et al.; Bedoin et al.; Cirelli et al.; Cumming et al.; Hannon et al.; Lense and Dykens; Teie). Among those, one theoretical contribution raised the intriguing possibility that an individual's fetal environment may already affect his future rhythmic repertoire (Teie). Two contributions tested rhythmic abilities in infants ranging from 7 to 15 months of age, with a focus either on beat perception (Cirelli et al.) or speech meter and grouping (Abboub et al.). A corpus-based approach investigated rhythmic regularities in children's songs and finds a connection between rhythms in song and non-song speech features (Hannon et al.). Two contributions focused on the interaction between musical beat and language in 9 year old children with specific language impairments (Bedoin et al.; Cumming et al.). Finally, Lense and Dykens tracked rhythmic abilities over the lifespan in a sample of 74 children and adults affected by Williams syndrome.

## Cross-cultural evidence

The study of rhythm in speech and music is increasingly adopting a global, cross-cultural perspective (Abboub et al.; Bekius et al.; Polak et al.; Roncaglia-Denissen et al.; Teie). The field seems to be expanding beyond learners of English as first language or musically-enculturated Westerners. In speech, three contributions explored the relationship between different languages or learning a foreign language, and rhythmic capacities (Abboub et al.; Bekius et al.; Hannon et al.; Roncaglia-Denissen et al.). In music, the focus is on biologically-driven rhythmic universals (Teie) and experiments involving cross-cultural comparisons (Polak et al.).

## EEG and frequency tagging

Along a methodological dimension, empirical papers adopted three alternative approaches: corpus analyses, behavioral experiments or brain imaging/electrophysiology. It is interesting to note that all experimental papers in this issue that employed EEG also adopted a frequency-tagging approach (Celma-Miralles et al.; Cirelli et al.; Teki and Kononowicz), rather than a grand-average ERP method (but see Henry et al., 2017 for a note of caution).

## Music, speech, and syntax

The relationship between music, language, and speech continues being of great interest in the scientific community. This continued interest is found also in the papers in this issue (Bedoin et al.; Bekius et al.; Cumming et al.; Geambaşu et al.; Norton and Scharff; Ravignani et al.; Roncaglia-Denissen et al.). In particular, one paper investigated how beat keeping and phonological patterning are related (Bekius et al.). Another study focused on recursion, a topic of great debate in linguistics and showed how human adults are sensitive to recursive structures in rhythmic patterns (Geambaşu et al.).

## Modality

Modality-specificity and domain-specificity were also explored in this issue (Celma-Miralles et al.; Matthews et al.; Richter and Ostovar; Su). Findings about rhythm in vision (Celma-Miralles et al.; Su) and movement (Su) suggested that some circuits for rhythmic timing may coincide across modalities.

## Rhythm in interaction

Complementary to meticulously controlled individual experiments, rhythm can be investigated by taking a more holistic approach, and probing rhythmic behaviors in interaction (Benichov et al.; Gamba et al.; Hartbauer and Römer; Lense and Dykens; Richter and Ostovar; Woolhouse et al.). Vocal coordination behavior in groups of primates (Gamba et al.), songbirds (Benichov et al.), and insects (Hartbauer and Römer) can offer insights for human interactional timing. Connections between internal rhythms and group behaviors can be investigated in healthy adults (Richter and Ostovar; Woolhouse et al.) and individuals with specific syndromes affecting musicality and sociality (Lense and Dykens).

## Dance

Three papers discussed rhythm from the perspective of dance (Richter and Ostovar; Su; Woolhouse et al.). Rhythm and dance should be thought as a tightly connected pair (Richter and Ostovar), which can be empirically investigated (Su) and shed light on other aspects of cognition (Woolhouse et al.).

## Quantitative models

Two papers in the issue were devoted to mathematical and computational modeling (Forth et al.; Jadoul et al.). These approaches are complementary. On the one hand, rhythm and timing can be investigated using top-down abstract models (Forth et al.). On the other hand, different aspects of speech timing can be statistically modeled with different degrees of precisions and assumptions made (Jadoul et al.).

## Animal research

This issue also contains ample evidence on rhythm from a comparative approach (Benichov et al.; Dufour et al.; Gamba et al.; Hartbauer and Römer; Hoeschele and Bowling; Norton and Scharff; Ravignani et al.; Rouse et al.; Spierings and ten Cate; ten Cate et al.). Songbirds continue to be a particularly often-used model species in the study of rhythm (Benichov et al.; Hoeschele and Bowling; Norton and Scharff; Spierings and ten Cate; ten Cate et al.). For instance, important advances have been made by confirming how the subjective “feeling of rhythm” experienced when listening to a songbird has a quantitative, isochronous counterpart in the animal's song (Norton and Scharff). Rhythmic behaviors in two primate species were also explored in this issue. These works examined either the closest primate to humans, the chimpanzee (Dufour et al.) or one of the phylogenetically farthest group, the lemur (Gamba et al.). This suggests that some components of human rhythmicity may be due to evolutionary homology (common descent from our last common ancestor with chimpanzees) while other traits to analogy (convergent evolution in man and singing lemurs). A taxonomic group emerging as particularly promising for future rhythm research is the pinnipeds, which features harbor seals, sea lions, and walruses (Ravignani et al.; Rouse et al.).

## General timing and others

Other papers discussed general issues related to timing and time perception (Rajendran et al.; Sameiro-Barbosa and Geiser; Teki; Teki and Griffiths). Two contributions tested general aspects of the relationship among timing, rhythm and cognitive functions (Rajendran et al.; Teki and Griffiths). A theoretical paper reviewed neural entrainment mechanisms (Sameiro-Barbosa and Geiser). Finally, Teki provided a useful overview of timing papers since 2000, ordering them by number of citations, so to identify community trends and overall research interests.

## Rhythm in other journals since late 2015

Since the launch of this Frontiers Research Topic, a number of publications on rhythm have appeared in other journals. Among those, some strands are particularly relevant to research in the evolution of rhythm. Far from attempting a comprehensive overview, we mention these papers and summarize some of them below.

### Evolutionary hypotheses for rhythm origins

Some review papers have properly focused on the evolutionary origins of musical rhythm and animal species showing human-like rhythmic traits (Bannan, 2016; Iversen, 2016; Wilson and Cook, 2016). Bannan (2016) provided a recount of Charles Darwin's thoughts on music and how he thought human musicality may have emerged via sexual selection. Iversen (2016) summarized and compared many evolutionary hypotheses on the origins of rhythm in humans. Wilson and Cook (2016) discussed which animal species are capable of synchronizing to a beat, either spontaneously or after being trained, and how this evidence relates to evolutionary hypotheses. Some of these evolutionary hypotheses on music and rhythm have been tested via genetics (Mosing et al., 2015), behavioral experiments (Miani, 2016), electrophysiology (Bouwer et al., 2016) or animal comparative work (ten Cate et al.; van der Aa et al., 2015).

### Speech rhythm and comparative anatomy of vocal tracts

In the evolution of speech, several studies have shown how vocal tracts in non-human primates are more flexible than previously thought. Other primates' vocal tracts are capable of producing a human-like range of vowels (Fitch et al., 2016; Boë et al., 2017) and consonants (Lameira et al., 2015, 2016, 2017). The overall conclusion is that the complexity of human speech, including its rhythmical nuances, must have neural, rather than morphological, bases (Ravignani et al., 2014b; Fitch et al., 2016; Belyk and Brown, 2017).

### The social roots of rhythm

The relationship between rhythm and sociality has seen a steady increase in research and has probably been the most investigated topic over the last 2 years (Large and Gray, 2015; Yu and Tomonaga, 2015; Ellamil et al., 2016; Gebauer et al., 2016; Greenfield et al., 2016; Moore et al., 2016; Reddish et al., 2016; Rennung and Göritz, 2016; Schirmer et al., 2016; Tunçgenç and Cohen, 2016; Wallot et al., 2016; Bishop and Goebl, 2017; Chang et al., 2017; Cirelli et al., 2017; Hannon et al., 2017; Knight et al., 2017; Mogan et al., 2017; Murphy and Schul, 2017; Rorato et al., 2017; Myers et al.). Common foci are the relationship between synchronization and prosociality (Gebauer et al., 2016; Reddish et al., 2016; Rennung and Göritz, 2016; Tunçgenç and Cohen, 2016; Cirelli et al., 2017), and different forms of rhythmic behaviors in interaction (Large and Gray, 2015; Ravignani, 2015; Yu and Tomonaga, 2015; Ellamil et al., 2016; Gebauer et al., 2016; Greenfield et al., 2016; Moore et al., 2016; Schirmer et al., 2016; Wallot et al., 2016; Murphy and Schul, 2017).

### Speech, music, and prosody

Another topic of broad interest centers on the relationship between speech, prosody, and music (Toro and Nespor, 2015; Vanden Bosch der Nederlanden et al., 2015; Chang et al., 2016; Filippi, 2016; Frühholz et al., 2016; Kotz and Schwartze, 2016; Schwartze and Kotz, 2016; Weidema et al., 2016; Carr et al., 2017; Ding et al., 2017; Spierings et al., 2017; Toro and Hoeschele, 2017). An intriguing hypothesis is that speech prosody may be the “missing link” between music and language (Filippi, 2016) or that music and language may be preceded by musical prosody (Fitch, 2013; Honing, 2017). This may inform us on early proto-musical and proto-linguistic behaviors in our early hominid ancestors.

### Cultural evolution and cognitive biases

Rhythm seems to be slowly overcoming the classical nature-nurture debate that actually is built on a false dichotomy. Along these lines, recent research has focused on the cultural evolution of musical rhythm and perceptual priors (Savage et al., 2015; Trehub, 2015; Hansen et al., 2016; Le Bomin et al., 2016; Ravignani et al., 2016; Fitch, 2017; Jacoby and McDermott, 2017). Statistical universals found in musical rhythms all over the world (Savage et al., 2015) can emerge via the combined effect of human cognitive biases and cultural transmission (Ravignani et al., 2016). Interestingly, these biases seem at least partly modulated by enculturation (Jacoby and McDermott, 2017).

### The evolution of dance

The field of musical rhythm is increasingly expanding to encompass the scientific study of dance (Ellamil et al., 2016; Fink and Shackelford, 2017; Fitch, 2016; Laland et al., 2016; Ravignani and Cook, 2016; Su, 2016). Only in 2016, three papers have introduced conceptual frameworks for the evolutionary study of dance (Fitch, 2016; Laland et al., 2016; Ravignani and Cook, 2016). We believe the field would benefit from connecting these theoretical frameworks with recent empirical findings on dance (Ellamil et al., 2016; Su, 2016).

### Timing and time perception

The science of timing and time perception has been a major research area in the last century. After a less active period, this field is again experiencing an increase in research efforts. A whole special issue of Current Opinion in Behavioral Sciences was recently devoted to “Time in perception and action” (Meck and Ivry, 2016). In addition, a “Timing Research Forum” was established in 2016, to spur and connect research on timing and time perception across disciplines.

### Measuring rhythm

Finally, new methods to model (van der Weij et al., 2017) and measure rhythmicity have been proposed, either quantitatively from data (Daniele, 2017; Malisz et al., 2017; Ravignani, 2017b; Ravignani and Norton, 2017) or as a test battery on human participants (Dalla Bella et al., 2016).

## Final considerations

Similarly to other fields, the study of the evolution of rhythm must build on a tight integration of experiments, theory, and modeling. Ideally, empirical observations of rhythm in music and speech are first recorded in the field. Observations are then contrasted to generate testable hypotheses. Based on these hypotheses, experiments on linguistic and musical rhythm are performed. Experimental factors and variations can encompass sensory modalities, ages, and animal species, to name a few, in order to address questions about domain-specificity, development, and evolutionary phylogeny. Finally, experimental insights should be integrated via synthetic modeling. The advantage of models is that they generate predictions that are quantitatively testable. Following these predictions, new empirical observations should be collected and compared, continuing the incremental loop of scientific investigation.

This journal issue contains novel empirical findings and state of the art reviews of hot topics in each discipline. We hope it will be useful as a reference volume on the evolution of rhythm cognition. Combining well-established findings and novel results on the evolution of rhythm, it should serve as an introductory reference for newcomers, a source of novel findings for researchers more familiar with one of the areas, and an interdisciplinary overview of progress in neighboring disciplines.

All contributions discussed so far show the many sides of rhythm. From this volume, rhythm emerges not as a monolithic concept, but as a multifaceted phenomenon for research. We hope that exciting future research will be ignited by this multifaceted display of rhythm across domains and species.

## Author contributions

All authors listed, have made substantial, direct, and intellectual contribution to the work, and approved it for publication.

### Conflict of interest statement

The authors declare that the research was conducted in the absence of any commercial or financial relationships that could be construed as a potential conflict of interest.
